# Supplementary figures and images for: Reduction of antisense transcription affects bovine leukemia virus replication and oncogenesis
Source: PLoS Pathog. 2024 Nov 7;20(11):e1012659. doi: 10.1371/journal.ppat.1012659 (PMC11575825; doi:10.1371/journal.ppat.1012659)

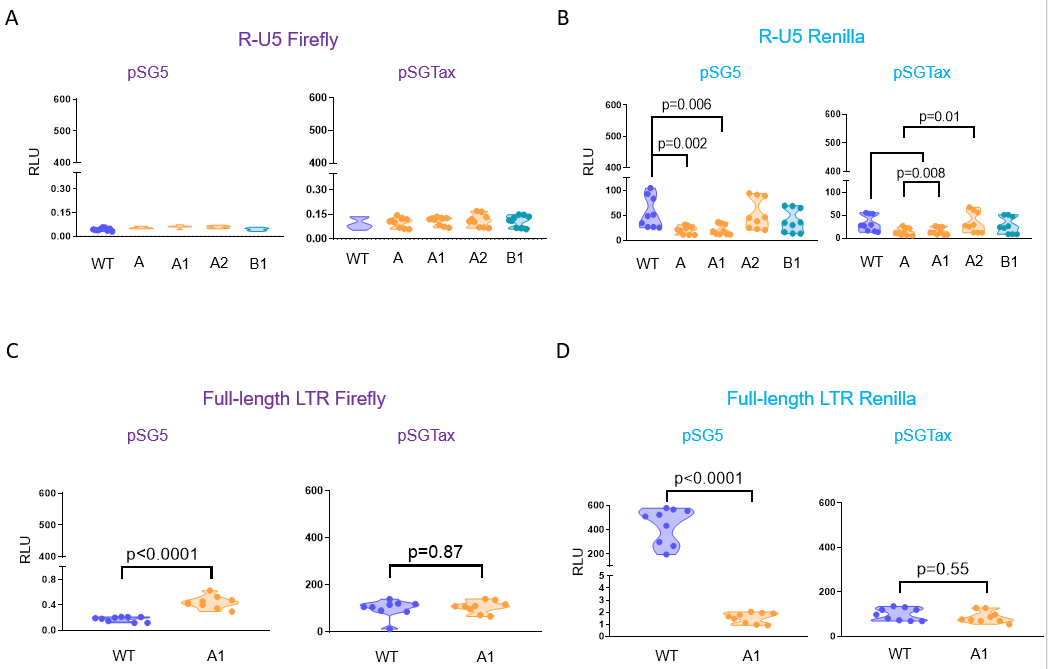

Supplement: S1 Data — Luciferase activities promoted by the minimal (R-U5, A-B) and full-length LTR (C-D) in OVK cells. Reporter plasmids (WT, A, A1, A2, B1) and vectors expressing either mock (pSG5) or the viral transactivator Tax (pSGTax) are described in Fig 1B. Twenty-four hours after transfection of OVK cells, Firefly (A) and Renilla (B) luciferase activities were measured and normalized to the mean luminescence of the pSGTax + pLTR samples, arbitrarily set to 100. RLU: relative luminescence units. Data results from at least three independent experiments. p-values were calculated according to Mann-Whitney tests. (TIF) [file ppat.1012659.s001.tif]

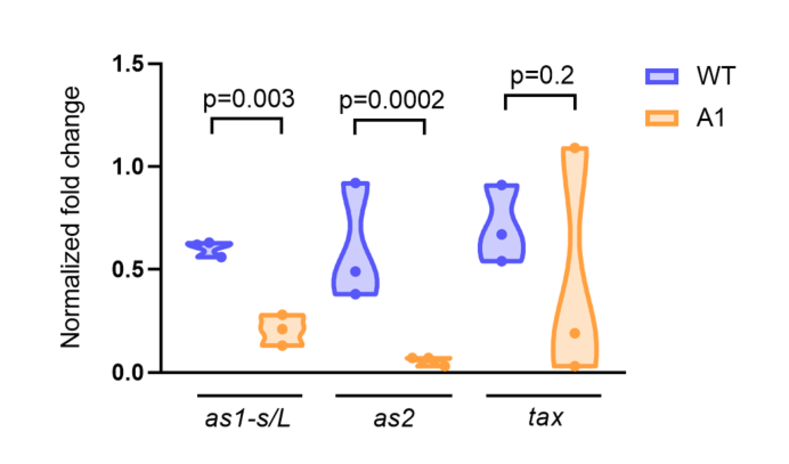

Supplement: S2 Data — HEK293FT cells transfected with either pBLV344 (WT) or pBLV344-A1 (A1) were cocultured with CC81 cells for 24 hours. After extraction and RT-qPCR, tax, as1-s/L and as2 RNAs were quantified by the ΔΔCT method using ß-actin as housekeeping gene. Data resulting from three independent experiments were normalized to the means of the WT samples, arbitrarily set to 1. p-values were calculated according to two-tailed Wilcoxon signed rank test. (TIF) [file ppat.1012659.s002.tif]

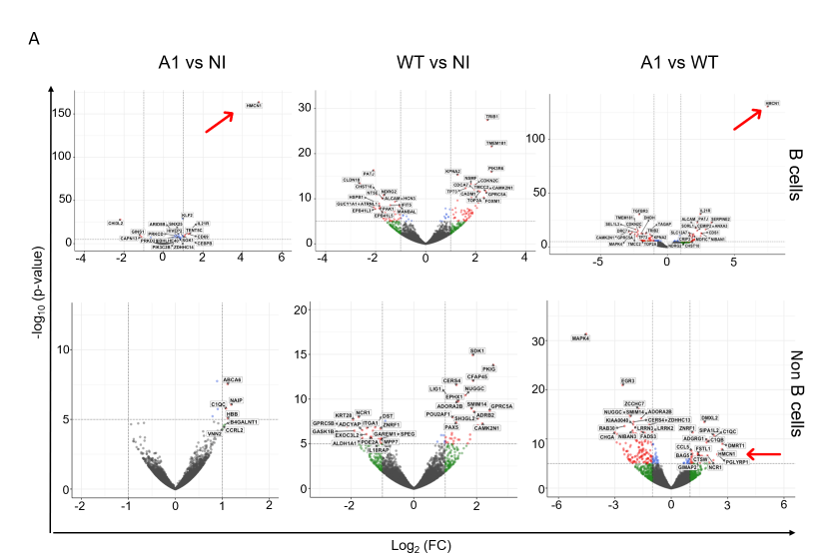

Supplement: S3 Data — Volcano plots were generated using the EnhancedVolcano (version: 1.16.0) and ggplot2 (version 3.3.3) R packages. The x and y axes correspond to log2(fold-change) (FC) and -log10(p-value), respectively. Cut-off values were defined as p-adj < 0.05 and |Log2FC| > 1. NI means not-infected and WT is wild-type. (TIF) [file ppat.1012659.s003.tif]

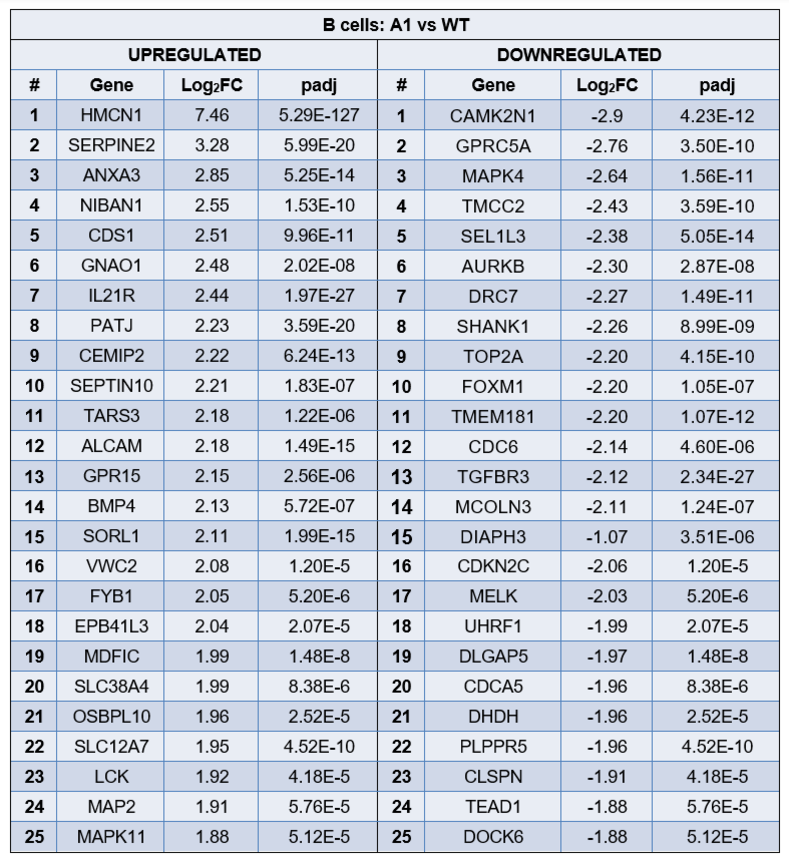

Supplement: S4 Data — (A) Top 25 list of significantly upregulated genes ranked by Log2(FC). (B) Top 25 list of significantly downregulated genes ranked by Log2(FC). (TIF) [file ppat.1012659.s004.tif]

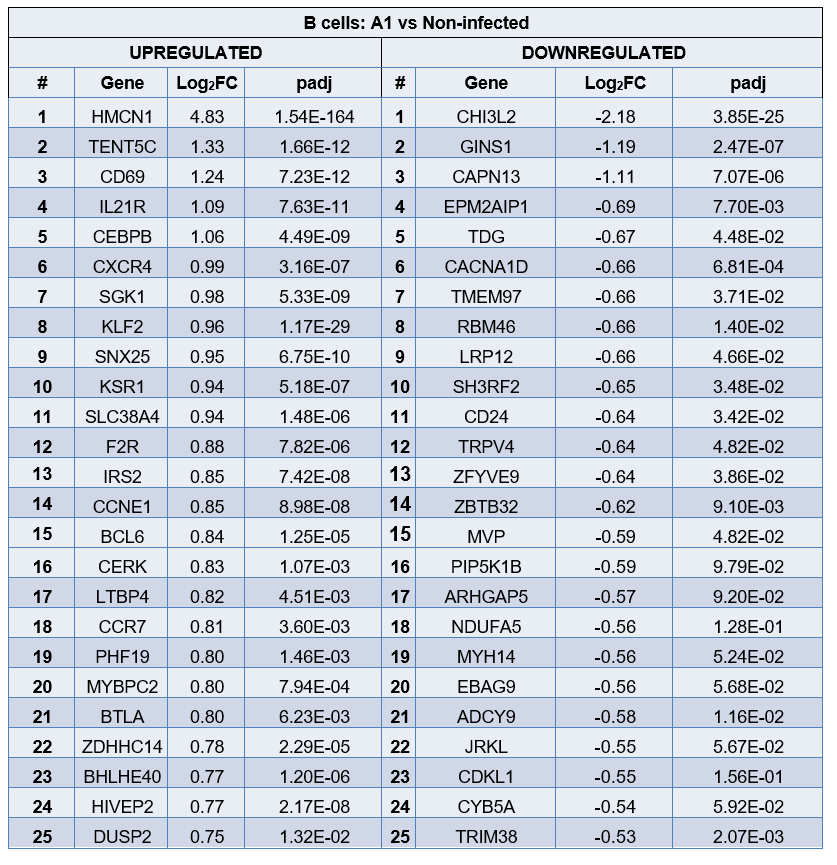

Supplement: S5 Data — (A) Top 25 list of significantly upregulated genes ranked by Log2(FC). (B) Top 25 list of significantly downregulated genes ranked by Log2(FC). (TIF) [file ppat.1012659.s005.tif]

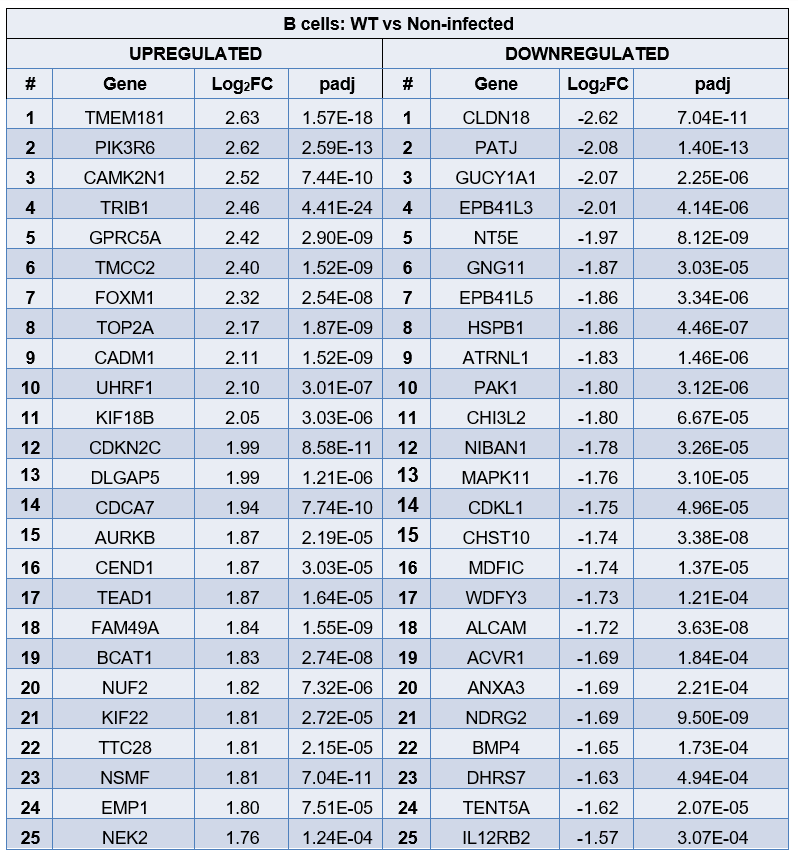

Supplement: S6 Data — (A) Top 25 list of significantly upregulated genes ranked by Log2(FC). (B) Top 25 list of significantly downregulated genes ranked by Log2(FC). (TIF) [file ppat.1012659.s006.tif]

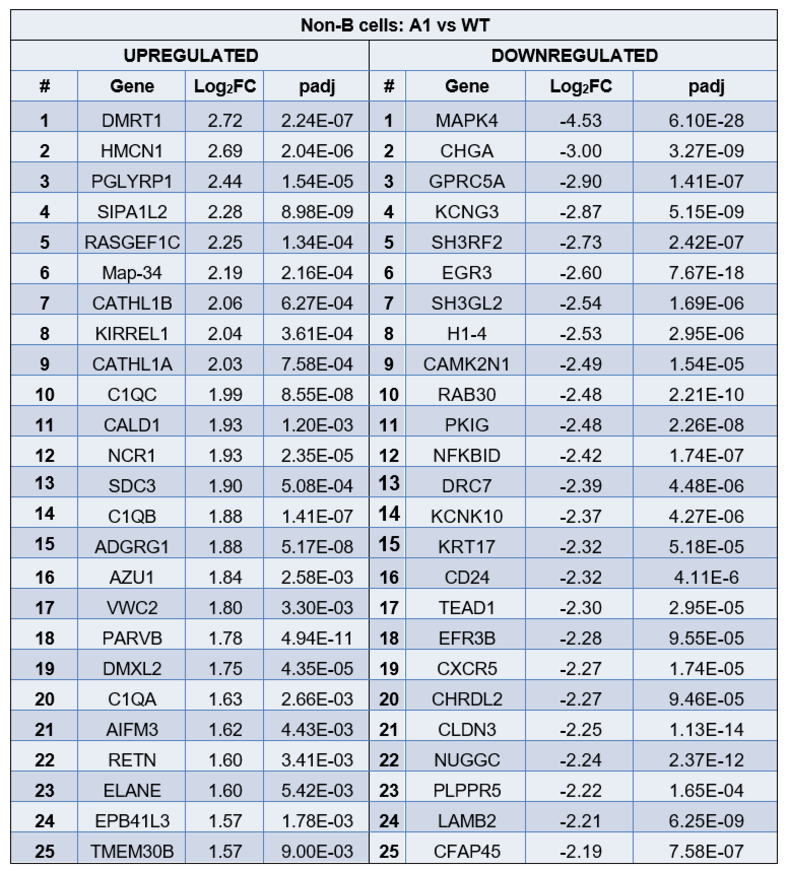

Supplement: S7 Data — (A) Top 25 list of significantly upregulated genes ranked by Log2(FC). (B) Top 25 list of significantly downregulated genes ranked by Log2(FC). (TIF) [file ppat.1012659.s007.tif]

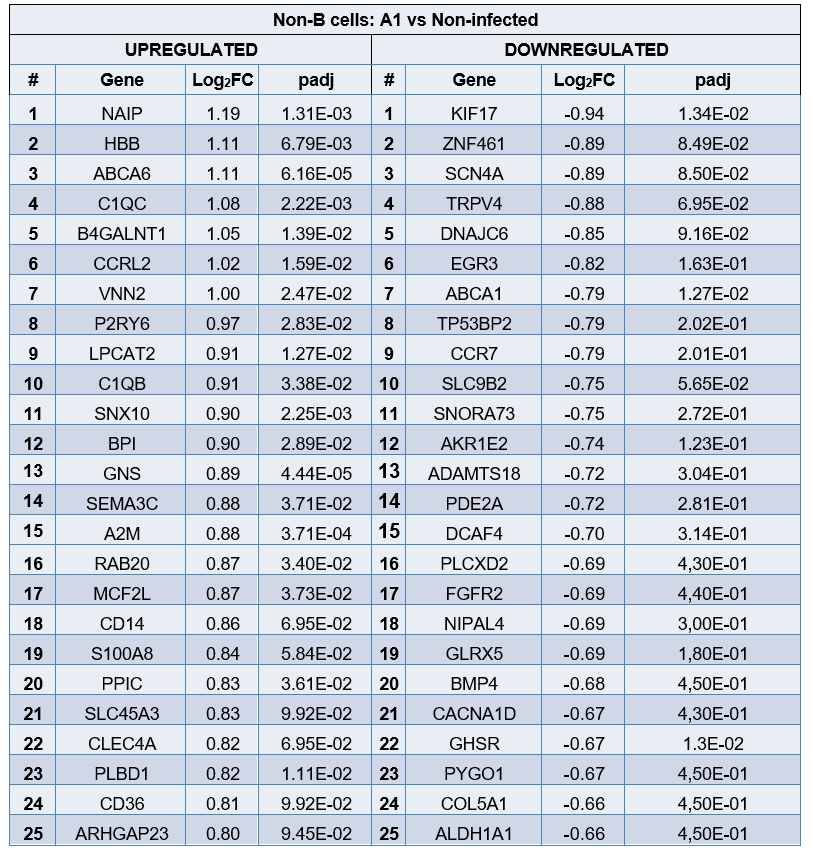

Supplement: S8 Data — (A) Top 25 list of significantly upregulated genes ranked by Log2(FC). (B) Top 25 list of significantly downregulated genes ranked by Log2(FC). (TIF) [file ppat.1012659.s008.tif]

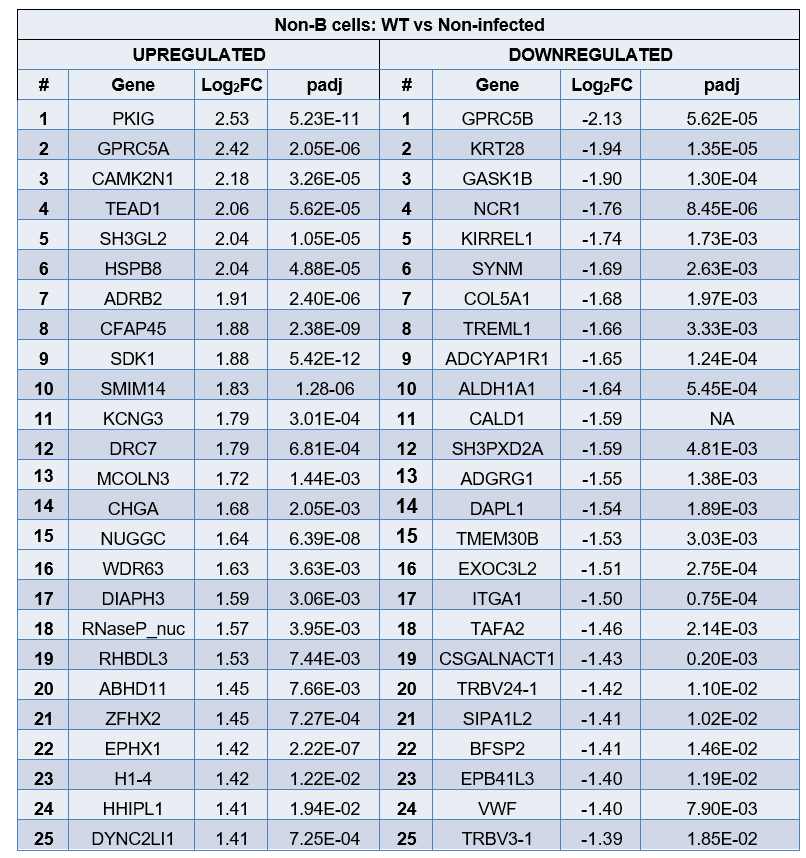

Supplement: S9 Data — (A) Top 25 list of significantly upregulated genes ranked by Log2(FC). (B) Top 25 list of significantly downregulated genes ranked by Log2(FC). (TIF) [file ppat.1012659.s009.tif]

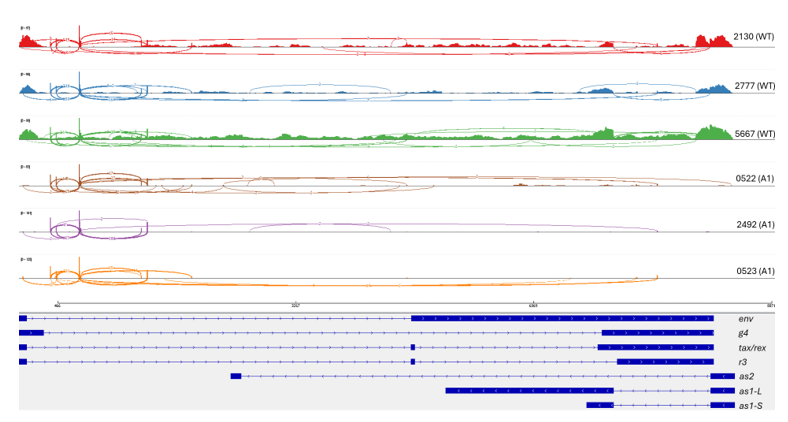

Supplement: S10 Data — RNA-Seq data was aligned on the genomic RNA sequence of the BLV344 provirus using STAR. The jsdbOverhang19 parameter was set at 19. Sashimi plots were generated for each sheep infected with the pBLV344 (WT) or pBLV344-A1 (A1) provirus using the Integrative Genomic Viewer desktop application (IGV_2.18.2). The minimum junction coverage was set at 2. Splicing of the as1-S/L RNA was detected for the three sheep inoculated with the WT. Due to low number of reads for the sheep inoculated with pBLV344-A1, the splicing pattern of the A1 virus was indeterminate. (TIF) [file ppat.1012659.s010.tif]

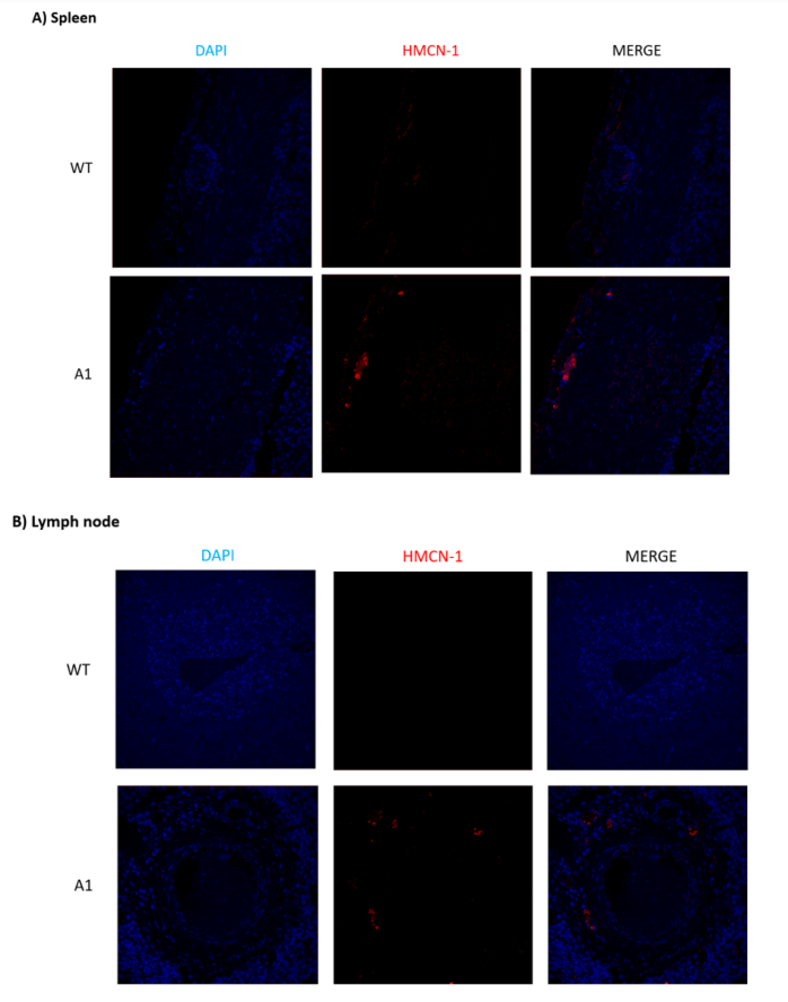

Supplement: S11 Data — Spleen (A) and lymph node (B) sections from WT and A1-infected sheep were stained with DAPI (nuclei in blue) and labelled with anti-HMCN1 antibody combined with an AlexaFluor 647 conjugate (in red). Slides were imaged with a Leica Stellaris confocal microscope. (TIF) [file ppat.1012659.s011.tif]

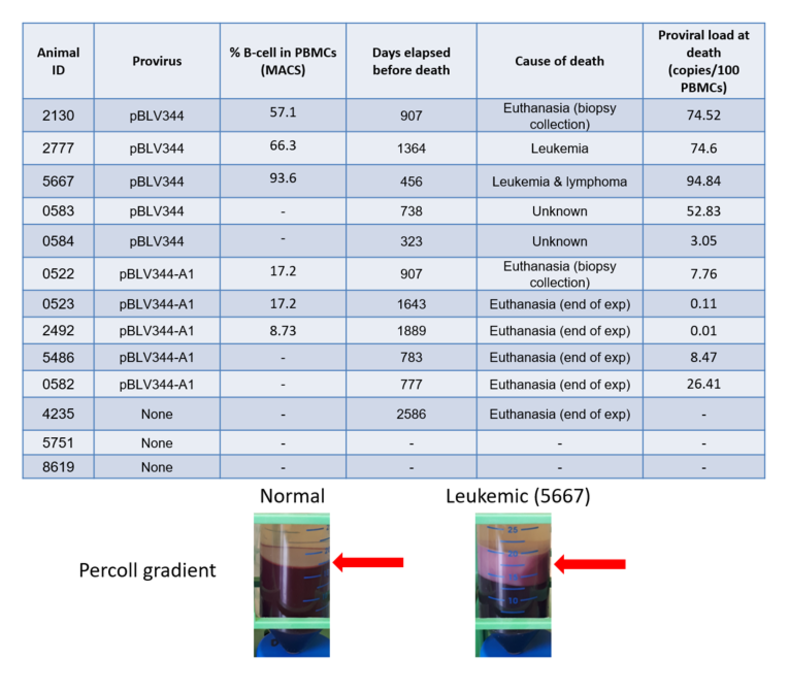

Supplement: S12 Data — Sheep of the Vendéen breed were inoculated with mock or WT/A1 proviruses and examined at regular intervals to evaluate pathogenesis. Upon blood collection, PBMCs were separated by Percoll-based gradient centrifugation. The percentages of B-cell in the PBMCs were determined by flow cytometry using an anti-sIgM antibody (1H4). After DNA extraction, proviral loads (in copies /100 PBMCs) were quantified by qPCR. The death resulted from leukemia/lymphoma, causes unrelated to BLV infection (unknown) or euthanasia for biopsy samplings (end of experiment). The pictures illustrate normal and leukemic blood after PBMC centrifugation. (TIF) [file ppat.1012659.s012.tif]

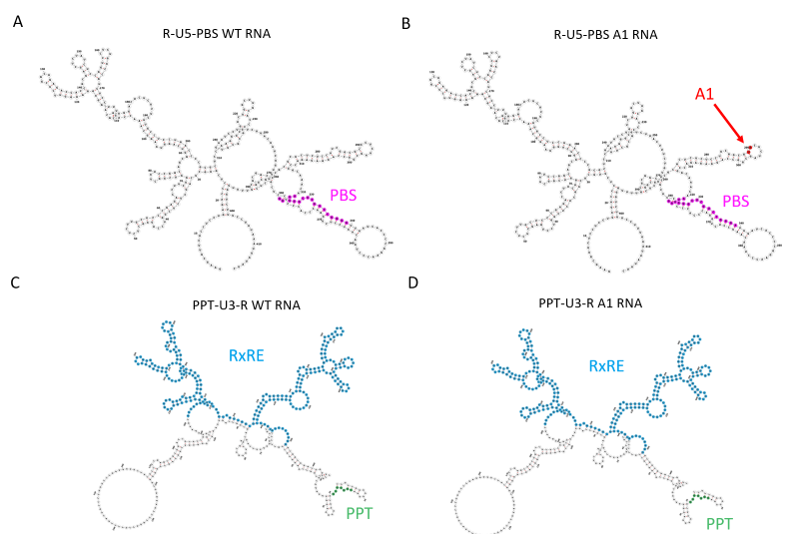

Supplement: S13 Data — (A) Structure prediction for the R-U5 region of the WT RNA were made using the RNAfold WebServer by the Vienna RNA Websuite. The Primer Binding Site (PBS) is represented in purple. (B) Corresponding structure of the A1 RNA. The red arrow points to the A1 mutation. (C) Structure prediction of the U3-R region of the WT RNA. The Rex responsive element (RxRE) is represented in blue and the Polypurine tract (PPT) is colored in green. (D) Corresponding prediction of the A1 RNA folded structure. (TIF) [file ppat.1012659.s013.tif]

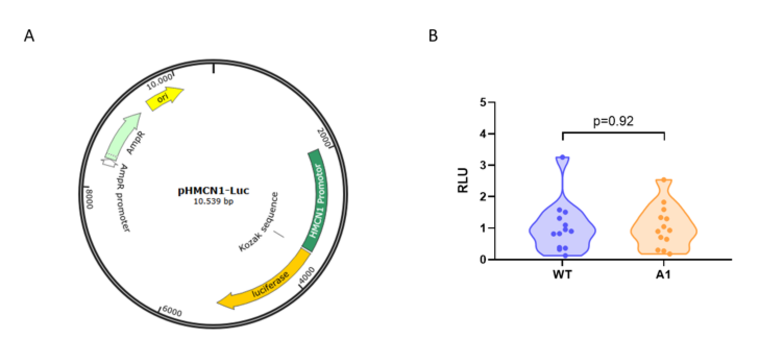

Supplement: S14 Data — (A) The pHMCN1-Luc reporter plasmid contains a 1570bp fragment overlapping the Hmcn1 gene promotor region (corresponding to the 64722376–64723946 location chromosome 12 of the Ovis Aries reference genome Oar_v4.0) cloned upstream of the humanized Luc2 Firefly luciferase gene. (B) Luciferase activities measured twenty-four hours after co-transfection of either pBLV344 or pBLV344-A1 plasmids. Firefly luciferase activities were normalized to the mean luminescence of the pSGTax samples, arbitrarily set to 1. Data results from of at least 10 independent experiments. p-values were calculated according to the Mann-Whitney test. RLU: Relative Luminescence Units. Ori: Origin of replication. AmpR: ampicillin resistance gene. (TIF) [file ppat.1012659.s014.tif]

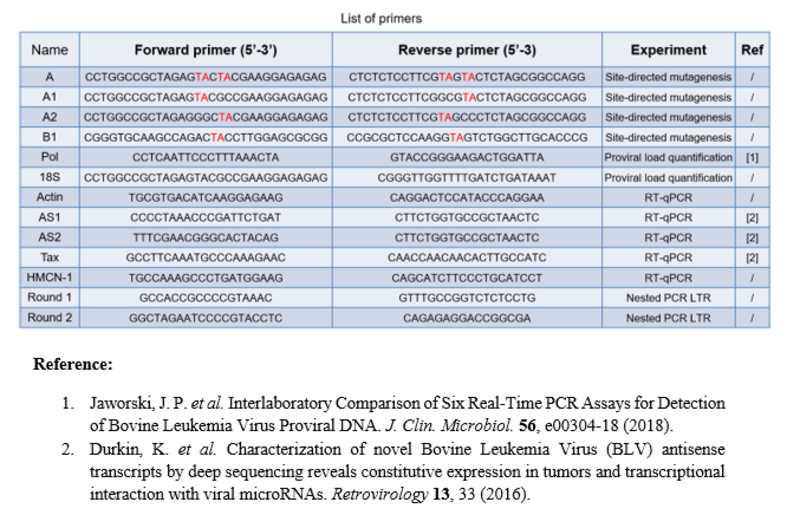

Supplement: S15 Data — (TIF) [file ppat.1012659.s015.tif]
