# Supplementary material for: Reduction of antisense transcription affects bovine leukemia virus replication and oncogenesis
Source: PLoS Pathog. 2024 Nov 7;20(11):e1012659. doi: 10.1371/journal.ppat.1012659 (PMC11575825; doi:10.1371/journal.ppat.1012659)
Supplement: Attachment — Submitted filename: Response PLoS Pathogens.docx [file ppat.1012659.s016.docx]

**Reviewer #1 (major)**

1a. *"... Regarding luciferase assays with a series of mutations for both UTR (Fig.1), promoter activity of the full-length LTR was assayed with A1 mutation only (Fig. 1F&G). To confirm the findings with R-U5, the other mutants should be evaluated similarly in the full-length LTR assay..."*

As requested, we performed additional experiments by introducing the A, A2 and B mutations in a full-length LTR. Luciferase reporter assays are now included in **Figure 1F and 1G**.

1b. *"... In addition, in OVK cells, when pSGTax was used (Supple Fig1.C&D), no significant difference was found in both LTR assays. The authors need to discuss why the difference was observed between 293FT and OVK cells..."*

The difference between the two cell lines is now discussed on **page 14**.

1c. *"... Also, the use of the name “A1” mutation should be avoided in the abstract..."*

The term "A1" has been deleted from the abstract.

2. *"...In terms of Fig.2B-2C, did co-cultured cells show reduced antisense transcriptions similarly to 293FT cells?..."*

To address this question, antisense transcription was determined by RT-qPCR in cocultures of HEK293FT and CC81. The RNA levels of as1-S/L and as2 expressed by the A1 mutant were reduced compared to the wild-type. The pattern of viral RNA expression was thus similar in HEK293FT cells cultured or not with CC81. The data are now included in **Supplementary Data 2**.

3a. *"...In Fig.5, although PVLs were decreased in both lymph nodes and spleen in the infected sheep with A1 mutant (Fig. 5C&D), Ki67 results were different in lymph nodes and spleen (Ki67 was more elevated in spleen with A1 mutant). Only this finding is at odds with the other results..." . "...The finding of decreased PVL but increased proliferative capacity in the spleen needs more detailed study. The authors also discuss the correlation between BLV and HTLV-1. However, as is well-known, the aggressive phenotype of ATL markedly increases the amount of HTLV-1 provirus compared to carriers and chronic/smoldering ATL..."*

There is indeed an apparent discrepancy that needs to be better explained. The data shows that there is no correlation between the proviral loads and proliferation determined by KI67 immunochemistry. The most straightforward interpretation is that most KI67-positive cells are not infected by the A1 mutant in the spleen and in lymph nodes. It is thus likely that these cells are T-lymphocytes that are activated by antiviral immunity. As supported by a number of previous reports, BLV infection is associated with an increase in T cell proliferation as a marker of an active antiviral immune response. Experiments based on intravenous injection of CFSE and BrdU would precisely quantify the cell kinetics in vivo. In BLV-infected sheep, antiviral immunity is centrally controlled by the spleen because splenectomy accelerates the onset of leukemia/lymphoma. On the other side, provirus-carrying B cells undergo active proliferation to persist, accumulate and ultimately induce leukemia in sheep. Similarly, the HTLV-1 induced Adult T-cell leukemia/lymphoma (ATLL) and HTLV-associated myelopathy / tropical spastic paraparesis (HAM/TSP) are also characterized by active proliferation of provirus-carrying cells. This is now better explained in the manuscript on **page 16-17**.

4. *"...In terms of survival analysis (Fig. 5H), are all observed events in wild-type infected sheep the development of lymphoma or leukemia?..."*

We agree that the survival analysis of Figure 5H was unclear. Therefore, we deleted panel H of Figure 5 and included additional information in **Supplementary data 12**.

**Reviewer #2 (major)**

1a. *"...It is not so clear why A1 mutation affects antisense but not sense transcription. Luciferase assay support the idea, but it is not clear if that is the case as whole provirus. More information about how they selected and introduce certain pattern of mutations (Figure 1B)..."*

As schematized in Fig.1A, the promoters for sense and antisense transcription are located in different regions of the LTR (respectively U3 and R-U5). It is thus possible to affect antisense transcription without modifying sense transcription. The mutations were targeted to the 2 transcription initiation sites of antisense RNAs (A, A1 and A2 in the BRE and B in the MTE/DPE). The GG>TA and GC>TA mutations are designed to disrupt the interaction of the promoter element to the RNA polymerase (TFIIB). This is now clarified on **page 7**.

1b. "...Viral transcriptome pattern from with RNA-seq data can be shown on IGV. That would be useful information if A1 mutation would change splicing pattern of proviral transcript..."

Because the proviral loads were strongly reduced in sheep inoculated with the A1 mutant, the amount of viral RNAs was also particularly low. Due to the low number of sequencing reads, the splicing pattern of the A1 mutant was indeterminate. However, the IGV confirmed that RNA loads were reduced in A1 PBMCs compared to WT and did not reveal any significant alteration of splicing events (**Supplementary data 10**).

2a. "...*How did they normalize dose of virus to inoculate each sheep?..."*

The protocol used for inoculation was described in a number of publications from the laboratory (*e.g.*, Willems 1992). All sheep were inoculated with the same amount (20μg) of wild-type and A1 proviruses.

*2b. "... How was the lymphocytosis in wild type and A1 mutant infected sheep?..." "...They enriched B cells for RNA-seq analysis. How was the proportion of B cells in the PBMCs ?..."*

The percentages of B cells in the PBMCs are now indicated in **Supplementary data 12**. We also provide information about the proviral loads at death.

2c. *"...Figure 3B; They showed PVL as copy number/ug DNA. I recommend to show copy number/100 PBMCs as usual..."*

We have now indicated the number of copies / 100 PBMCs.

2d. *"...Figure 3C; How did they normalize RNA loads? PBMCs or B cells or infected cells?
Since PVL was very low in A1 sheep, as2 transcripts of A1 sheep may be higher than that of wild type if we normalized by PVL..."*

Since the RNA loads are very low in sheep inoculated with the A1 provirus, the normalization to the proviral load generates inconsistent results. Therefore, data resulting from at least three independent experiments were normalized to the means of the WT sample, arbitrarily set to 1.

2e. *"...Figure 3E; It is good to know percentage of p24 positive cells. Then we can know % of infected cells in the PBMCs. Then we can have some idea how many copy of provirus per infected cells in this sheep model..."*

It should be mentioned that the percentages of p24-positive cells do not reflect the number of infected cells. Indeed, the proportion of provirus-carrying cells able to express p24 is animal-dependent and varies with time. Therefore, the proviral loads reflect better the number of infected cells.

2f. "...Figure 3G; It is informative if they show sense and antisense BLV transcript level in this plot..."

A volcano plot that included the sense and antisense transcripts was unclear. Therefore, we propose to represent the BLV transcripts in an IGV plot (**Supplementary data 10)**, as requested in comment 1b.

2**g**. *"...Figure 4E: What total means in the calculation?..."*

The term "total is inadequate and has been deleted.

3a. *"...Figure 3H. If infectivity is very low, the effect of A1 mutation on B cell transcriptome should not be mediated by cell intrinsic way but indirect effect. ... It would be more convincing if the authors would show effect of antisense BLV transcript on Hmcn-1 expression..."*

As requested, we evaluated the effect of the antisense RNAs on *Hmcn1* transcription. Luciferase-based reporter assays with a plasmid controlled by the Hmcn-1 promoter did not reveal significant difference after transfection of A1 and WT proviruses, suggesting an indirect mechanism ongoing inside infected cells. This is now included in **Supplementary data 14**.

3b. *"...It is not clear how Hmcn-1 expression change induced phenotypic changes between wild type and A1 mutant BLV. If I understand correctly, logical flow is like below.
A1 mutation -> decreased BLV antisense RNA -> increased Hmcn-1 expression in B cell -> low viral persistence and pathogenesis. I’d like to ask authors to make the logic clear..."*

As requested, we now better explain the rationale on **page 15-16**.

**Reviewer #3 (major)**

*"...Figure 5B & 5A (L237): Additional data that Ki-67+ cells are T cells rather than B cells would be desirable. In general, a higher provirus level in the early stages of infection seems to lead to a stronger T-cell response. However, the result that the cell proliferation in spleen is rather induced in the A1 group with low provirus levels seems to contradict this..."*

We fully agree with this interpretation that is now better explained on **page 16**.

*"... Figure 5G (L249): If DNA samples are available, PVL data up to the end of the study period should be shown. Some individuals not marked dead (cross) (especially group A1) have PVL data ending in the middle of the study period..."*

The PVL data at the end of the study is now added in **Supplementary data 12**. In Figure 5G, some animals are not marked dead because they were inoculated later. The PVL data are shown until the latest available quantification. This is now specified in the figure legend.

**Reviewer #1 (minor)**

"*Page 7, Line 136 “OVK” should be spelled out and explained when it is used firstly in the text*."

This has been corrected.

*"Page 10, Line 198 “analyzes” should be an error in writing."*

Corrected

*"Page 11, Line 217. For Fig. 4F, incucyte live-cell imaging should be described in more detail in the text to make it easier for readers to understand."*

We added a description of the experiment on page 11.

**Reviewer #2 (minor)**

1. *"Figure 1F, Figure 2C; It is not well explained why A1 mutation showed different effect on sense transcription. If they show more control data about transfection efficiency is similar between wild type and A1 mutant, the data will be more convincing."*

Transcription efficiencies were controlled routinely between wild type and A1 mutant.

2. *"Figure 2C and 2D; How can we explain why Tax expression was low but infectivity was high in A1 mutant?*

Tax expression was not significantly different between the wild type virus and the A1 mutant (Figure 2C). Infectivity assay based on OVK-U3Luc cells increases significantly only by 1.7 fold (Figure 2D). We interpret this observation with caution, particularly because cell fusion is unaffected (Figure 2F).

3. *"Line 55; Please correct sentence “Gene expression was affected.”*

Corrected

4. *"Line 59-60; The author mentioned ‘These findings may offer valuable insights into understanding the relevance of antisense transcription in the context of human T-cell leukemia virus (HTLV-1).’ There are similarities as well as difference between HTLV-1 and BLV. I am wondering what is the exact value of this research for HTLV-1 infection?"*

The idea is that understanding the BLV model may be useful in a perspective of comparative leukemia.

5. *"There are many typo and grammar issues*"

Corrected

6. *"Figure 4; image is not so clear. Please use better ones."*

Corrected

7. *"Figure 4E; The phenomenon we are discussing in this study is In B cells. Why the authors use OVK cells?"*

The reason is that there is no available ovine B cell line that has good transfection efficiency.

8. *"Line 240; Again, it is not clear how the authors performed special distribution of provirus. What is the total PVL value. Did they extract genomic DNA from whole body and calculated total PVL in the sheep?"*

We now better explained this point on page 22.

9. *"Figure 5; how many sheep they analyzed?"*

10 sheep were analyzed. 5 in the WT group, 5 in the A1 group. Additionally, 3 healthy (non-infected) sheep were analyzed for RNA-seq. A list of all the sheep is now included in **Supplementary data 12.**

10. *"Line 288; Is this sentence correct?"*

We meant that the A1 molecular clone optimally fulfils the different characteristics required to address the role of antisense transcription: i.e., reduced antisense transcription and similar sense transcription.

11. *"Line 656; No description of Incucyte live imaging in the text/methods"*

The description of the Incucyte experiment is now included on page 11

**Reviewer #3 (minor)**

*" L207: Regarding the cellular localization of Hemicentin-1 (Figure 4B), it seems that A1-infected PBMCs have a stronger localization to the plasma membrane than A1-infected PBMCs. If this is correct, what is the link between the change in localization and the effect on cell migration?"*

A detailed analysis of hemicentin localization did not reveal a significant difference at the cellular level between A1 and WT.

*" L249: In Figure 5G, there are some individuals whose plots are interrupted in the middle of the study, but according to Figure 5H, they survived until the end of the study. Is it correct? Since there are discrepancies in the data interpretation, please provide a list of the animal information (animal ID, breed, sex, age, date and cause of death or withdrawal from the study, clinical and necropsy findings) as Supplementary Data., then identify the individuals on the graphs (Figure 5F and G)." "L250: Is it confirmed that the cause of death of all sheep that died during the observation period was due to the onset of a tumor? If not, please indicate the incidence of tumors."*

The PVL data are shown until the most recent available quantification. This date corresponds to the death of the sheep. Because additional sheep have been inoculated later, some data were not available. For clarification, a table is added in **supplementary data 12**.

*"L258: I strongly agree that a comparative virological approach using a BLV-infected sheep model would be beneficial in understanding the pathogenesis of HTLV-1-related disease. However, whether the findings on BLV antisense transcription in this study can be extrapolated to disease mechanisms induced by antisense transcription of HTLV-1 needs careful validation and discussion. In a previous study by the authors (Ref. 23), they reported that HTLV-1 HBZ RNA inhibits 5'LTR promoter activity and abolishes proviral sense transcription, thus facilitating HTLV-1 entry into latent infection and immune evasion. This is at odds with the results obtained in this study for BLV and does not allow us to consider the same RNA-dependent epigenetic mechanism of antisense transcription of BLV and HTLV-1. How about adding a little more on this point in the discussion?"*

Epigenetic regulation is discussed based on reference 23 that describes the role of the HBZ RNA in regulation of HTLV-1 transcription.

*"L303: As discussed by the authors, they do not have a detailed explanation of why antisense transcription suppresses BLV replication in vivo. The authors propose three hypotheses about it (L303-306). A more detailed explanation of each hypothesis would be helpful for the reader's understanding."*

A more detailed explanation has now been included.

*"L316: The authors indicate that TGFβ and MAPK may be involved in the induction of Hemicentin-1 expression in B cells. Please indicate references if any."*

References have been included.

*"L660 or Figure 4H: Please explain in the legend which cells you have determined as Hemicentin-1 positive cells or mark the immunostaining pictures."*

This has been done.
